# Effects of continuous visual feedback during sitting balance training in chronic stroke survivors

**DOI:** 10.1186/s12984-017-0316-0

**Published:** 2017-10-16

**Authors:** Laura Pellegrino, Psiche Giannoni, Lucio Marinelli, Maura Casadio

**Affiliations:** 1 Department Informatics, Bioengineering, Robotics and Systems Engineering, University of Genoa, Via Opera Pia, 16145 Genoa, Italy; 2 Department of Neuroscience, Rehabilitation, Ophthalmology, Genetics, Maternal and Child Health (DINOGMI), University of Genoa, L.go Daneo, Genoa, 16132 Italy; 3 Department of Neuroscience, Ospedale Policlinico San Martino, L.go R. Benzi, Genoa, 16132 Italy

**Keywords:** Visual feedback, Trunk control, Posture, Stroke survivors, Center of pressure, Motor learning

## Abstract

**Background:**

Postural control deficits are common in stroke survivors and often the rehabilitation programs include balance training based on visual feedback to improve the control of body position or of the voluntary shift of body weight in space. In the present work, a group of chronic stroke survivors, while sitting on a force plate, exercised the ability to control their Center of Pressure with a training based on continuous visual feedback. The goal of this study was to test if and to what extent chronic stroke survivors were able to learn the task and transfer the learned ability to a condition without visual feedback and to directions and displacement amplitudes different from those experienced during training.

**Methods:**

Eleven chronic stroke survivors (5 Male - 6 Female, age: 59.72 ± 12.84 years) participated in this study. Subjects were seated on a stool positioned on top of a custom-built force platform. Their Center of Pressure positions were mapped to the coordinate of a cursor on a computer monitor. During training, the cursor position was always displayed and the subjects were to reach targets by shifting their Center of Pressure by moving their trunk. Pre and post-training subjects were required to reach without visual feedback of the cursor the training targets as well as other targets positioned in different directions and displacement amplitudes.

**Results:**

During training, most stroke survivors were able to perform the required task and to improve their performance in terms of duration, smoothness, and movement extent, although not in terms of movement direction. However, when we removed the visual feedback, most of them had no improvement with respect to their pre-training performance.

**Conclusions:**

This study suggests that postural training based exclusively on continuous visual feedback can provide limited benefits for stroke survivors, if administered alone. However, the positive gains observed during training justify the integration of this technology-based protocol in a well-structured and personalized physiotherapy training, where the combination of the two approaches may lead to functional recovery.

## Background

In our daily life we maintain different postures and automatically adjust our postural responses before starting voluntary movements [1, 2]. Following a stroke, some of these abilities are often compromised [3]. Several studies reported an increased sway during quiet standing [4], an uneven weight distribution with increased weight bearing on the unaffected limb in stance [5], a decreased weight-shifting ability [6], and abnormalities in postural responses [3, 7, 8]. For stroke survivors, good postural control is important to support the weak side and to reduce the effects of the altered postural tone [9, 10]. The impairments in sitting balance arise mainly because of muscle weakness, loss of dexterity, sensory deficits, and tendency to adopt compensatory strategies for avoiding threats to balance [11].

A major focus of rehabilitation programs is to improve balance and mobility for greater functional independence. Visual feedback is largely used in rehabilitation to improve the control of standing or sitting posture and to train the ability to shift weight by moving the entire body or the trunk [12, 13]. A number of devices used in the clinical practice provide training based on the feedback of a cursor on a computer screen, controlled by the position of the Center of Pressure (CoP) or of the Center of Mass (CoM) [1, 14, 15].

Several studies showed that stroke survivors who received rehabilitation treatments based on visual feedback of their weight distribution on both feet or about their CoP or CoM position, regained better standing symmetry than those who received conventional physical therapy [16–18] or therapies designed to offer tactile and verbal cues regarding postural symmetry [4]. Sackley and Lincoln [19] demonstrated that this improved stance symmetry was also associated with an increased ability to perform functional tasks. Lee et al. [20] showed that in chronic stroke survivors, training with visual feedback improves both static and dynamic sitting balance as well as visual perception. However, a Cochrane review [21] and two reviews on standing balance in stroke survivors [22, 23] highlighted a limited evidence of benefits of this type of training. According to these reviews, different studies reported small improvements [24–26] with a limited long-term retention and no significant benefits compared to conventional physical therapy [21, 27].

With respect to sitting balance, there is evidence that following a stroke, the muscles of the trunk are compromised [22, 28], resulting in poor trunk control during voluntary trunk and limbs movements [29, 30]. However, few studies address the problem and the effects obtained with exercises based on visual feedback are still unclear; see [23] for a review.

In the last few years, vision has been the feedback modality most intensively investigated in the context of optimizing augmented feedback for motor learning [31]. This line of research has revealed how visual feedback strategies can either facilitate [19] or impair motor learning [32, 33].

Schmidt et al. [34] demonstrated that concurrent feedback - i.e. feedback provided continuously during motor task execution - can enhance performance in the acquisition phase, but the performance gains are lost in retention tests. This finding suggested that permanent feedback during acquisition lead to a dependency on that feedback.

Moreover, the control of posture involves vision, proprioceptive and tactile feedback, as well as vestibular input, and their sensorimotor integration [35]. Vision is a dominant form of feedback [15, 36–38], and one may argue that by focusing mostly on vision, we could reduce our attention on proprioceptive feedback [39], that is fundamental in postural control. Therefore, it is important to understand what we improve in terms of ability to control and correctly estimate the position of our body in space or to shift our weight, by focusing mainly on continuous visual feedback.

Our hypothesis is that postural training with continuous visual feedback is not effective at developing motor programs (i.e., feedforward control) that can be executed without reliance on feedback and applied to variety of operating conditions.

Thus, our specific goal was to understand if and to what extent it is possible to improve sitting postural stability of stroke survivors by training them to shift their CoP while providing them with continuous visual feedback of its position.

Here, we report an experiment where chronic stroke survivors seated on a stool were trained to guide a point representing their CoP in different directions toward a fixed distance from a resting neutral position. As they became increasingly able to control the motion of their CoP under visual guidance, we investigated the retention of this skill once the visual feedback was removed and the transfer of the learned ability to different directions as well as displacement amplitudes.

## Methods

### Subjects

We enrolled 11 chronic stroke survivors (5 Male - 6 Female, age: 59.72 ± 12.84 years) recruited among the outpatients of the Department of Neuroscience of Ospedale Policlinico San Martino, Genoa, Italy.

Clinical assessment of the stroke survivors was based on specific tests for evaluating trunk control and balance: Berg Balance Scale (BBS) [40], Trunk Impairment Scale (TIS) [41] and Nottingham Sensory Assessment Scale (NSA) [42]. The subscale for kinesthetic sensation of the NSA scale was used to determine proprioceptive function. BBS and TIS tests have been validated for use in the stroke population and have been used to characterize balance deficits [41, 43].

The exclusion criteria were: BBS < 25, TIS < 7, severe hypovision (visual acuity with corrective lenses less than 1/10), cognitive disorders such as neglect (Albert’s Test, [44]), inability to understand simple instruction (Mini-Mental State Examination, MMSE > 24, [45]) and inability to discriminate colors. All subjects had no severe aphasia or problems of visual integrity and all were able to clearly see the visual feedback provided in the computer monitor. Table 1 summarizes the stroke subjects’ demographic information and scores in the clinical scale.

**Table 1:** Demographic data and clinical scores for stroke survivors

	Sex	Age	PH	E	SL	DD	BBS	TIS	NSA
		(ys)				(ys)	(0–56)	(0–23)	(0–3)
P01	F	64	L	H	Right fronto-parietal prerolandic	9	50	11	2
P02	F	64	L	H	Right occipital	15	52	11	2
P03	F	43	R	I	n.a.	11	52	18	3
P04	F	39	R	I	Left basal ganglia, internal capsule and parietal lobe	9	54	19	2
P05	F	40	L	I	Right complete middle and anterior cerebral arteries	10	48	10	0
P06	M	61	R	H	Left basal ganglia	2	45	11	0
P07	F	65	R	I	Left basal ganglia, internal capsule and insula	2	27	11	1
P08	M	66	L	H	Right fronto-parietal	12	52	13	2
P09	M	71	L	I	Right posterior capsule	4	54	16	3
P10	M	69	L	I	Right paramedian pontine	8	40	13	2
P11	M	75	R	H	Left basal ganglia and internal capsule	13	26	9	0
Mean		59.72				8.63	45.45	12.90	1.54
SD		12.84				4.34	10.25	3.33	1.12

*PH* Paretic hand: (Right/Left), *E* Etiology, *I/L* Ischemic/Hemorrhagic, *SL* Site of lesion, *DD* disease duration (years), *BBS* Berg Balance Scale, *TIS* Trunk Impairment Scale, *NSA* Nottingham Sensory Assessment Scale (kinesthetic section), *n.a.* not available

The research conforms to the ethical standards laid down in the 1964 Declaration of Helsinki that protects research subjects. Each subject signed a consent form to participate the study that conforms to these guidelines and was approved by the local Ethical Committee (ASL 3 Genovese 09/04/2013). Moreover, all subjects consented to publish individual data.

### Set-up and protocol

**Fig. 1:**
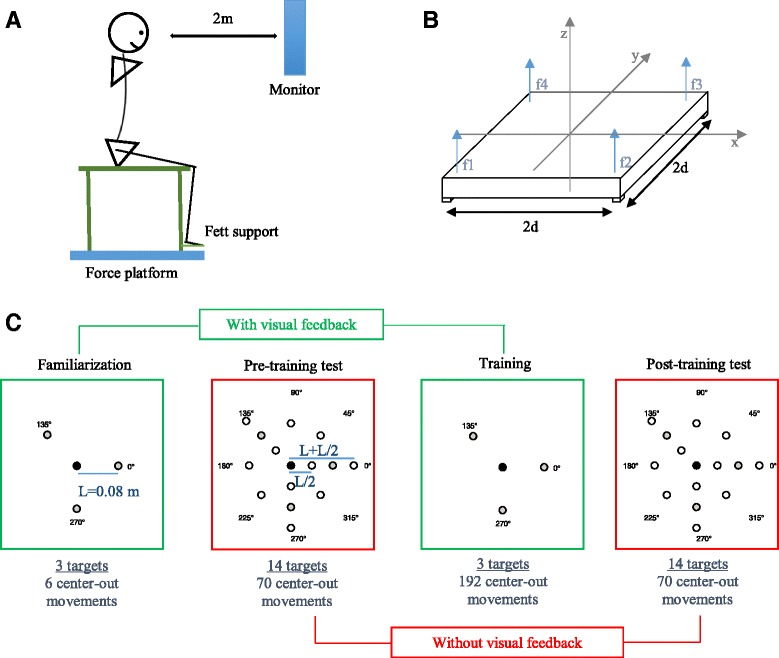
Panel **a**: Experimental set-up; Panel **b**: Schematic figure of the force platform with four load cells; f1, f2, f3 and f4 are the forces measured by each load cell. 2d =40 cm is the distance between two consecutive load cells. Panel **c**: Experimental protocol. The circles represent the peripheral targets’ positions on the computer screen; in black is the home target, in grey the three targets presented during the training phase and in white the targets presented only during the pre-training test and post-training test

Subjects were seated on a stool without back support and with the hands resting on their legs. The stool had a support for the feet and it was positioned on top of a custom-built force platform. The platform (50X50 cm surface) supported the entire body weight of the subjects (Fig. 1 panel a). The signals from four load cells positioned on the four corners of the platform (Fig. 1 panel b) were collected at 1 kHz by using Real Time Windows Target, Simulink, Mathworks.

The CoP coordinates were computed in real time by using the following equation:

$$ {\mathrm{x}}_{\mathrm{CoP}}=\frac{{\left({\mathrm{f}}_2+{\mathrm{f}}_3-{\mathrm{f}}_1-{\mathrm{f}}_4\right)}^{\ast}\mathrm{d}/2}{\sum_{\mathrm{i}=1}^4{\mathrm{f}}_{\mathrm{i}}} $$

$$ {\mathrm{y}}_{\mathrm{CoP}}=\frac{{\left({\mathrm{f}}_3+{\mathrm{f}}_4-{\mathrm{f}}_1-{\mathrm{f}}_2\right)}^{\ast}\mathrm{d}/2}{\sum_{\mathrm{i}=1}^4{\mathrm{f}}_{\mathrm{i}}} $$

where f_*i*_ was the force measured by the *i* (*i* = 1 to 4) load cell and d (d = 40 cm) was the distance between two adjacent load cells.

The estimated CoP positions were mapped to the coordinate of a cursor (yellow circle, 5 mm radius) on a computer monitor, positioned two meters away from the platform at eye level. When the subject’s trunk was in the upright posture (no bending), the cursor was at the center of the screen. Targets were displayed as circles (1 cm radius) against a black background.

The scale factor between the CoP displacement and the monitor was adjusted to allow each subject to comfortably move the cursor over the entire screen (scale factor: 1.8 mean ± 0.2 SD; this determined a shift of the CoP in the range of four to five cm for a displayed cursor motion of length L = 8 cm). This calibration procedure was performed before the experiment started. Subjects were asked to move their trunk along the four cardinal directions and the intermediate diagonal directions. Then, the gain was set such that they were able to move their CoP without difficulties in all the workspace. This process ensured that all subjects could comfortably reach the entire task space independent of their individual ability and anthropometric characteristics.

At the beginning of the experiment, the experimenter instructed the subjects to hold their trunk in the upright position with a correct alignment of the spine, so as to keep the cursor in the central (home) position. The physical therapist and the experimenter controlled that this condition was satisfied by each subject at the beginning of each target set.

The experimenter asked participants to reach the targets in 2 s and to be as fast and as accurate as possible. The 2 s started when subjects left the starting position (i.e., distance of the cursor form its starting position > = target radius). We explained to the subjects that, in all phases, after these 2 s the color of the target would turn red, indicating that the time to reach the target elapsed.

The protocol consisted of four phases: familiarization, training, and pre- and post- training tests.

**▪ Familiarization.** The goal of this phase was to explain to the subjects how and to what extent they had to move for reaching the targets. During this phase, three targets (Fig. 1 panel c, grey targets) positioned at L distance (L = 8 cm) from the center of the computer screen were presented twice (6 center-out movements) and the cursor position was always displayed. The targets were presented in three different directions: 0, 135, and 270 deg. Two targets were located in the basic cardinal directions, i.e. one in the antero-posterior direction (Fig. 1 panel c; 270 deg., backward) and the other in the medio-lateral direction (Fig. 1 panel c; 0 deg). The third target was on purpose selected in a diagonal direction (Fig. 1 panel c; 135 deg., frontal-lateral direction). The main reason for this choice was that functional movements and relative shifts of the CoP have rarely components only in the sagittal or in frontal planes, but often they are in directions that required combinations of motion in both planes [46]. The shift at 0 deg. corresponded to the impaired side. The selection of the side for the lateral direction was based on the impairment rather than dominance, because we expect that the former influence the performance more than the latter.

**▪ Training**. The goal of this phase was to exercise movements of length L = 8 cm toward the three directions presented also in the familiarization phase (Fig. 1 panel c, grey target). During the training phase, subjects performed 8 target sets. In every set, the three peripheral targets were presented eight times in pseudo-random order, with the condition that each peripheral target was not presented again before all three targets were reached. Therefore, subjects performed a total of 8 * 3 * 8 = 192 center-out movements. A new peripheral target was not presented to the subject if the cursor was not in the central (home) position. This ensured that each cursor center-out motion started from the central target. Since we asked the subjects to minimize the error after 2 s from their movement onset, we explained to them that the color of the peripheral target would change from green to red after these 2 s. If the cursor reached the target before these 2 s, the target would not change color, i.e. would remain green. Instead, if the cursor was not inside the target after these 2 s, the target would turn red and it would remain red until the cursor reached the target. In both cases the target disappeared after the cursor stayed inside the target for 1 s. Then a new target would appear in the home position. Visual feedback of the cursor was suppressed for the first 2 s of the movement on 1/8 of the center-out trials. The order of presentation of the no visual feedback trials was pseudo-random, with the constraint that all peripheral targets were presented once without visual feedback in each movement set. The cursor disappeared as the new target appeared. The cursor re-appeared 2 s after it moved out of the home target. Then, if after these two seconds, the subjects did not reach the desired target, they should correct their error as in the other trials of the training phase, where the visual feedback of the cursor was available.

Subjects were aware of the presence of these no VF trials. This suppression of the VF was applied to test how subjects transferred the improvement in performance to the no visual feedback condition during the training phase.

▪ **Test.** The goal of the test phases was to test if and to what extent subjects were able to transfer performance improvement obtained with visual feedback training to conditions where they had to move (i) without visual feedback of the cursor and (ii) to different directions and (iii) to different displacement amplitudes (scaling-expansion) with respect to the training phase. To reach this goal we compared the performance without visual feedback in the pre-training and in the post-training tests. The pre-training and post-training test phases were identical and consisted of 5 target sets. In each target set 14 targets were presented in random order. Eight equi-spaced targets positioned at the trained distance (L = 8 cm) from the center, three along the directions presented both in the familiarization and in the training phase and five located along not trained directions. The other six targets were presented along the trained directions, but at different distances from the home position: three targets located at half of the trained distance from the center L/2 = 4 cm and three targets located L/2 further with respect to the trained distance (L + L/2 = 12 cm). Therefore, in the test phases subjects performed a total of 5 * 14 = 70 center-out movements.

The visual feedback of the cursor was always absent. In each movement-set the first peripheral target would appear only when the subjects were in the home position. In each trial, when a target was presented subjects had 2 s to reach it. After these 2 s, the color of the target turned red and subjects were instructed to stop moving their CoP. After 10 ms, this red target disappeared, a new target appeared and subjects could move again their CoP to reach the new target as described above. We carefully check in the data analysis that all subjects followed the instruction to stop when the time elapsed.

The position held when a new target was presented was considered as new starting position. The protocol alternated one of the 15 peripheral targets and the home target. Subjects could fail to reach correctly both the home target and the peripheral targets. This fact was taken into consideration in the data analysis (see below).

The experimental session lasted about 1 h. The protocol required a minimum of 2 min break between each phase and subjects were allowed resting when and as long as they needed. We video recorded all subjects while performing the experiment.

### Data analysis

We focused on the control signal - i.e. CoP - that determined the cursor motion. The cursor trajectories were sampled at 100 Hz and filtered by using a 6th order Savitzky-Golay filter with a time window of 170 ms (equivalent cut off frequency: ~11 Hz), which allowed us to estimate the first three time derivatives (speed, acceleration, and jerk). The movement onset was defined as the first time instant when the cursor speed exceeded a threshold (10% of peak speed) [47]. The movement termination was defined as the last time the cursor speed went below and remained under the same threshold for 1 s. Another important time point considered in the following analysis is 2 s after the cursor (visible or invisible) left the starting target. This was the time given to the subjects for the subject to reach the target. In all trials after these 2 s the color of the target turned red indicating that the time to reach the target elapsed. During training if the subjects had not correctly captured the target in these 2 s they could correct their error while the test phases, no corrections were allowed.

We analyzed the following performance indicators:

- Reaching error at 2 s (RE2): the distance between the cursor and the target calculated at the end of the first 2 s of the cursor movement. This measure accounts for movement accuracy (aiming) and speed [48], i.e. the error at 2 s decreases if a subject moves faster and in the correct direction.
  We also looked at the reaching error after 2 s in terms of extent error and directional error. We considered two vectors: one connecting the starting point of the cursor motion with the target, the other connecting the same starting point with the cursor position after 2 s of motion.
  The directional error was computed as the angle between these two vectors, while the extent error was computed as the difference between their lengths. We analyzed both the absolute error (average of the absolute values obtained in each trial) and the systematic error (average of the signed values obtained in each trial). The systematic extent error indicated if the subjects undershoot (negative values) or overshoot (positive values) the target.
  While in the trials with visual feedback, the starting point was always inside the home target, in the blind trials this might not be the case, i.e. the subjects might believe to be in the home target while they were not. In this case, they could not account for their initial shift and they would aim at peripheral targets located in a circle centered on their actual starting position (instead of the home position). To verify that the errors we observed were not mainly caused by this mismatch of the initial positions, we computed the direction and extent errors also toward target locations translated such that the center of the target space corresponded to the cursor starting position, i.e. the location of the cursor when the peripheral target appeared.
- Movement Duration: the time difference between movement onset and movement termination.
- Normalized jerk index (NJ): the root mean square of the jerk (third time derivative of the trajectory), normalized with respect to movement amplitude and duration [49]; this is a measure of smoothness for the cursor control. We computed this indicator considering (averaging) both the entire trajectory from movement onset to movement termination (JN) and the first 2 s of the movement from the movement onset (NJ2).

Finally, we verified if in the blind target sets the subjects were able to maintain correctly the central home position and if they improved this ability after training. This is particularly important because returning correctly to the home position reflects the ability to recognize and maintain a correct alignment of the spine without retroversion of the pelvis [50] and/or a lateral weight shift [51], problems often observed in stroke survivors.

We considered the position of the cursor when it had stopped moving and the subjects assumed to be in the home position, just before the appearance of a peripheral target.

To quantify the systematic and the variable errors of the shift with respect to this position, we used the following indicators, similar to those defined by Dukelow et al. [52]:

- Variability: this indicator was calculated by computing the standard deviation of the x and y coordinates of the cursor position for each target location and averaging these standard deviations across all target positions for the x (Var_x_) and y (Var_y_) coordinates. Then we combined both Var_x_ and Var_y_ values as follow (Var_xy_):

$$ {Var}_{xy}=\sqrt{Var_x^2+{Var}_y^2} $$

- Systematic shift: this indicator is the constant error between the position of home target and the position of the cursor. We computed the mean error between the position of the home target and the corresponding cursor positions for each target location and then we averaged the obtained values across all target positions. We computed this indicator for the x (Shift_x_) and the y (Shift_y_) coordinates and both values combined (Shift_xy_) as follow:

$$ {\mathrm{Shift}}_{\mathrm{x}\mathrm{y}}=\sqrt{{\mathrm{Shift}}_{\mathrm{x}}^2+{\mathrm{Shift}}_{\mathrm{y}}^2} $$

For both the variability and the systematic shift indicators, we considered the average values of the five target sets of the pre-training test and the five target sets of post-training test.

#### Statistical analysis

**Fig. 2:**
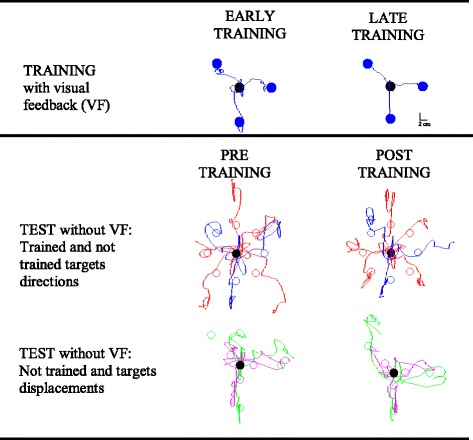
Cursor trajectories of a stroke survivor. The first row refers to the initial (first column) and final (second column) performance obtained in the training phase. The second and the third rows refer to the pre-training test (first column) and the post-training test (second column). Specifically, the second row shows the movement in the trained (blue lines) and not trained (red lines) target directions; the third row represents the movement for not trained target displacements

Statistical analysis was carried out within Statsoft environment (Statistica 7.1, Statsoft TULSA, USA). Repeated-measures ANOVA compared the performance measures across training periods, visual feedback conditions, target directions and displacements. Specifically, to test how performance changed during practice we ran a repeated measure ANOVA with two factors: training (first vs last movement set) and directions (0, 135, 270 degree; Fig. 2).

A post-hoc analysis (Fisher’s LSD test) was used to verify statistically significant differences among directions after repeated measures ANOVA.

To quantify if and how the learned abilities were transferred to the no visual feedback condition, (i) to different target directions and (ii) to displacement amplitudes, we ran a repeated ANOVA with two factors: training (PRE vs POST, i.e. pre-training test vs post-training test) and (i) directions (8 equi-spaced directions) or (ii) displacement amplitudes (target distance from the center: L/2, L, L + L/2), respectively.

We tested for sphericity using the Mauchly’s test; when the sphericity was violated we applied the Greenhouse-Geisser correction.

To specifically test for differences between the three trained and the five not trained directions as well as between the trained and the untrained displacement amplitudes in the post-training test we used planned comparisons.

Changes in performance within subject were tested by using the Student’s t-test for paired samples.

Also, for each condition, we verified if there were significant changes by comparing all the trials of the pre-training test with all the trials of the post-training test or comparing only the first three trials before and the first three trials after training.

Finally, we verified if there were significant changes between the pre-training test and the post-training test with respect to the variability and systematic shifts of the subject’s home position in the blind trials by using the Student’s t-test for paired samples.

Effects were considered significant at the *p* < 0.05 level.

## Results

### Performance during training with continuous visual feedback

All stroke survivors were able to improve their performance as a result of the training except P11; this subject had no statistically significant changes in performance for all the indicators at the beginning and at the end of the training. Since P11 did not change the performance during training with visual feedback, we couldn’t test the transfer of the improvement to other conditions. In the following we analyzed the changes in performance due to training only for the other ten stroke survivors. The stroke survivors had poor performance in the first block of training, as shown by the trajectories of a selected subject in Fig. 2.

**Fig. 3:**
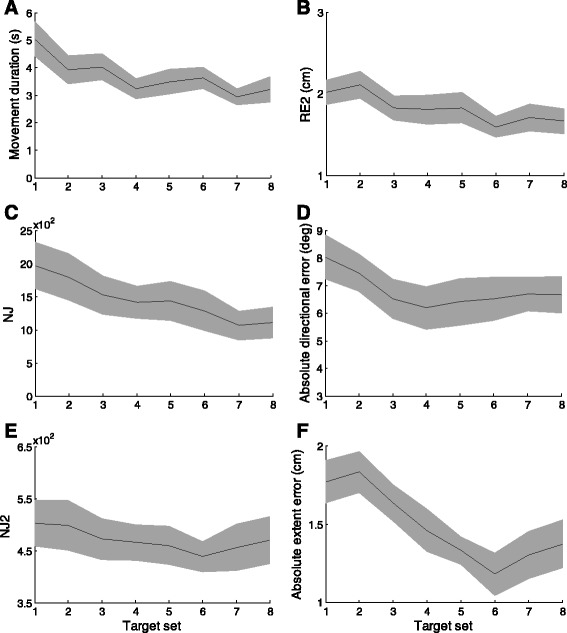
Changes in performance during training with visual feedback for movement duration (panel **a**), RE2 (panel **b**), NJ (panel **c**), absolute directional error (panel **d**), NJ2 (panel **e**) and absolute extent error (panel **f**). The back lines represent the mean value of each indicator. The shaded areas indicate the standard deviation

However, with training they improved duration, accuracy, and smoothness of the cursor trajectories (see Fig. 3 panel a: movement duration F(1,9) = 52.48, *p* < 0.001; panel B: RE2 F(1,9) = 14.67, *p* = 0.01; panel C: smoothness – NJ F(1,9) = 12.50, *p* = 0.01).

With respect to accuracy, the stroke survivors reduced significantly their absolute extent error (Fig. 3 panel f; training effect: F(1,9) = 19.82, *p* = 0.01). The systematic extent error highlighted a tendency of the population to slightly undershoot peripheral targets (beginning: −0.9 ± 0.3 SE cm; end of the training −0.8 ± 0.2 SE cm) and this strategy did not change significantly with training (F(1,9) = 0.92, *p* = 0.53).

In the first target set of the training, stroke survivors had a relevant absolute directional error, but most of them (7 subjects out of 10) did not improve with training (Fig. 3 panel d; training effect: F(1,9) = 2.90, *p* = 0.12).

The analysis of the smoothness showed that stroke survivors had a lower normalized jerk over the first 2 s of cursor motion (JN2: Fig. 3 panel e) with respect to the second part of the cursor trajectory (training effect: F(1,9) = 7.27, *p* = 0.03), i.e. the stroke survivors made few corrections in the first 2 s of motion although their trajectories had a relevant directional error. Then, they corrected the cursor position in the last part of the trajectory by using the visual feedback.

The performance of stroke survivors depended on the target direction in terms of duration (F(2,18) = 52.48, *p* < 0.0001), smoothness (NJ: F(2,18) = 12.5, *p* = 0.021) and accuracy of the cursor trajectories (RE2: F(2,18) = 6.91, *p* = 0.032; directional error: F(2,18) = 0.21, *p* = 0.81 and extent error: F(2,18) = 10.55, *p* = 0.041).

For the stroke survivors both the diagonal (135 degree) direction and the lateral (0 degree) displacement toward the impaired side were difficult to control. Thus, the backward direction was significantly easier to control than the other two directions in terms of duration (270 vs 0 deg.: *p* < 0.001, 270 vs 135 deg.: *p* < 0.001), smoothness (NJ, 270 vs 0 deg.: *p* = 0.04 and 270 vs 135 deg.: *p* < 0.001) and accuracy (RE2, 270 vs 0 deg.: *p* < 0.001 and 135 vs 0 deg.: *p* = 0.01, extent error: 270 vs 0 deg.: *p* = 0.001 and 135 vs 0 deg.: p = 0.03).

For all the indicators the rate of improvement was equal across directions despite the differences in the initial performance, i.e. there were not interaction effects between direction and training.

### Performance in the trials without visual feedback

**Fig. 4:**
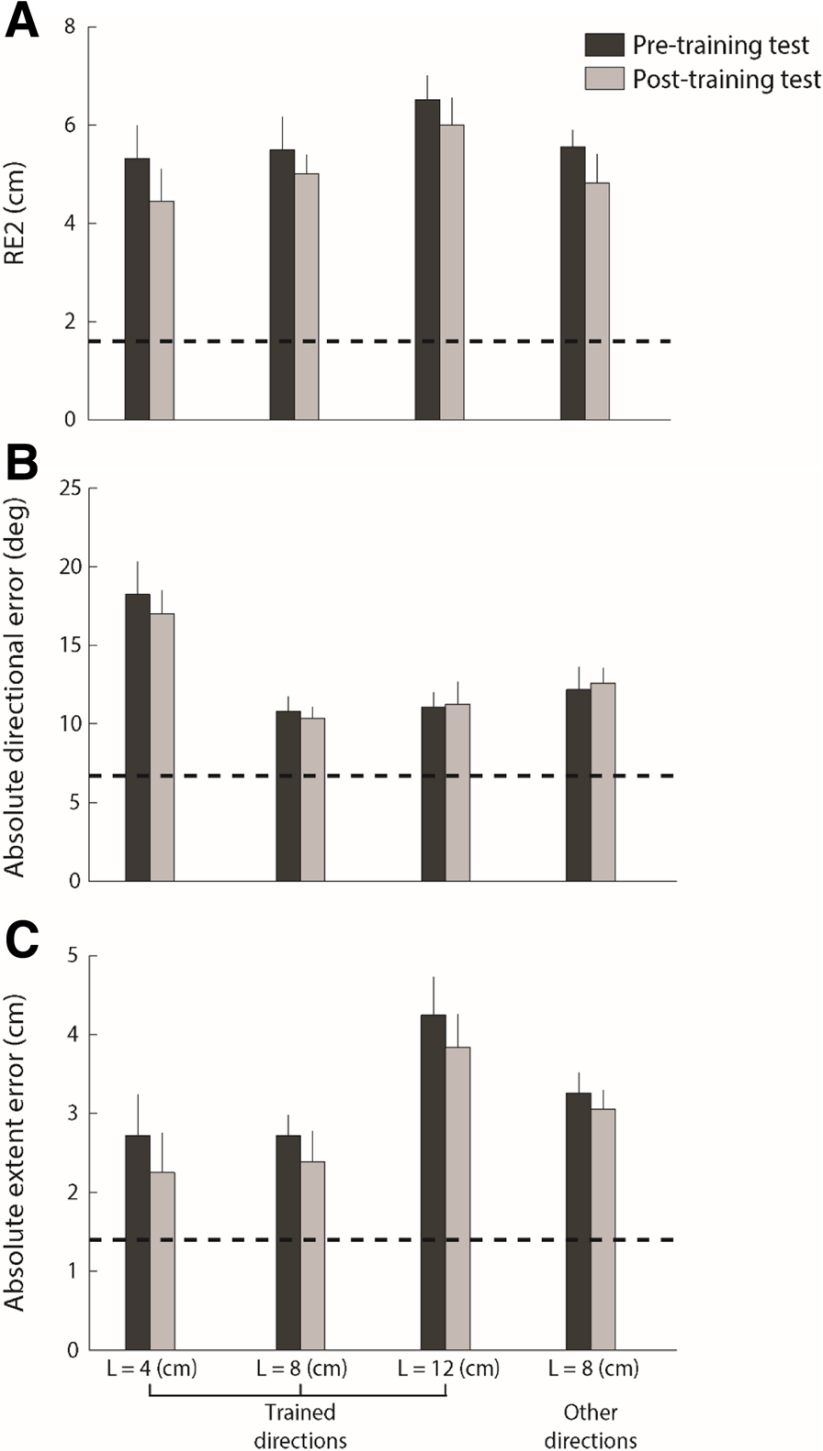
Each bar group represents the performance in the pre-training test (dark grey) and in the post- training test (light grey) for RE2 (panel **a**), absolute directional error (panel **b**) and absolute extent error (panel **c**), without visual feedback, for different displacements and directions of the targets as indicated in the horizontal axis. The height of the bars represents the mean value of the indicators; the error bars correspond to the standard error of the indicators. The dotted lines represent the mean value of the indicator during the last target set of the training phase with continuous visual feedback

We expected worse performance in post-training test with respect to the level they reached at the end of the training with visual feedback because of the absence of visual feedback, and indeed the accuracy was lower (see Fig. 4, panel a: RE2 F(1,9) = 70.97, *p* < 0.001; panel B: directional error F(1,9) = 11.98, *p* = 0.01; panel C: extent error F(1,9) = 29.85, *p* < 0.001). However, since the stroke survivors improved their performance in the visual feedback condition during training, to verify if they transferred to any extent the learned ability to the no visual feedback condition, we compared the performance in the pre- and post-training test. This comparison highlighted that most stroke survivors had evident difficulties to transfer to any extent the learned abilities to the no visual feedback condition, to different target directions, and displacement amplitudes. Specifically, in the post-training test, the RE2 displayed a trend of improvement with respect to the pre-training test, but without statistical significance neither in the trained targets nor in targets at the different distances or directions (Fig. 4 panel a; displacement amplitudes: F(1,9) = 8.57, *p* = 0.23 and targets’ directions: F(1,9) = 10.82, *p* = 0.42). The absolute directional error had no relevant changes in the training phase, thus we expected no improvement also in the post-training test (Fig. 4 panel b; displacement amplitudes: F(1,9) = 0.12, *p* = 0.73 and target directions: F(1,9) = 1.14, *p* = 0.31). However, the improvement obtained for the absolute extent error during training was not transferred to the test in absence of VF, neither for the different target amplitudes nor directions (Fig. 4 panel C; displacement amplitudes: F(1,9) = 2.27, *p* = 0.16 and targets’ directions: F(1,9) = 3.51, *p* = 0.19).

In the signed extent error, we observed that the stroke survivors in the absence of visual feedback overshoot the targets for L/2, L, L + L/2 distance (2.0 ± 0.2 SE cm, 2.4 ± 0.3 SE m and 3.1 ± 0.8 SE cm, respectively); but there was no significant improvement in the post-training test (1.9 ± 0.2 SE cm, 2.2 ± 0.2 SE cm and 2.9 ± 0.7 SE cm, respectively) with respect to the pre-training test (F(1,9) = 0.26, *p* = 0.70). Both in pre-training and post-training test phases, this error increased with increasing target distance. No improvement was also observed with respect to the different target directions (F(1,9) = 2.21, *p* = 0.83).

These results were also confirmed by the analysis of the no VF trials during training, where stroke survivors had no significant changes for any indicators, e.g. the RE2, the absolute extent error and absolute directional error were approximately equal in the first (5.1 ± 0.7 SE cm, 1.5 ± 0.1 SE cm and 5.87 ± 0.181 SE deg., respectively) and in the last training set (5.0 ± 0.9 SE cm, 1.6 ± 0.1 SE cm and 5.47 ± 0.253 SE deg., respectively).

**Fig. 5:**
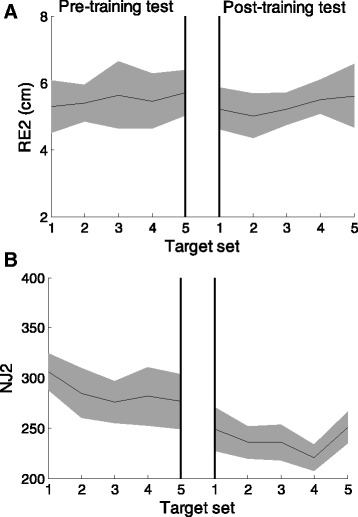
Change in performance during all targets sets of the pre-training test and of the post-training test for RE2 (panel **a**) and NJ2 (panel **b**) when the cursor moves in the trained directions. Dark black shaded areas indicate the standard deviation

The accuracy indicators were stable in the target sets of the pre-training test and post-training test phases for stroke survivors (Fig. 5 panel a). The NJ2 was the only parameter that decreased (Fig. 5 panel b) in the target sets without VF for the different target amplitudes (F(1,9) = 14.14, *p* = 0.02) and directions (F(1,9) = 31.49, *p* < 0.001).

#### Systematic shift and variability of the starting position

**Fig. 6:**
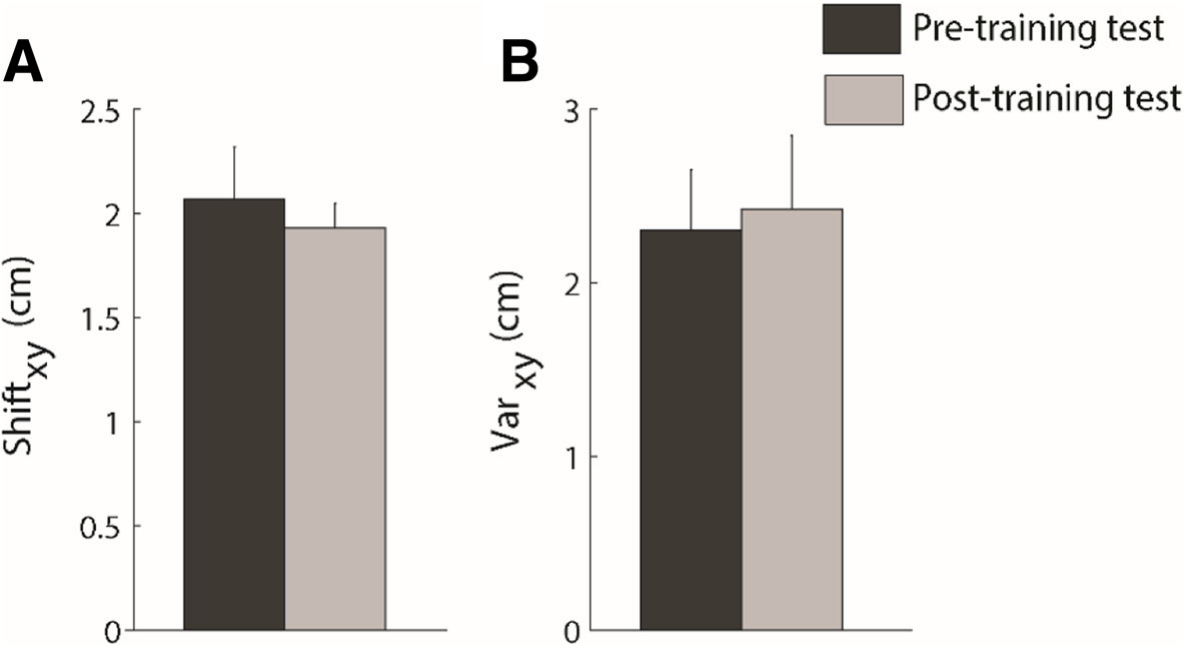
The bar group represent the systematic shift (panel **a**) and the variability (panel **b**) of the cursor positions when matching the home starting target in the pre-training test (dark grey) and in the post-training test (light grey). The height of the dark bars represents the mean value of the indicators; the error bars correspond to the standard error

Stroke survivors exhibited a systematic shift (Shift_xy_ – pre-training test: 2.0 ± 0.4 SE cm and post-training test: 1.9 ± 0.2 SE cm) and high variability of the starting position (Var_xy_ - pre-training test: 2.3 ± 0.2 SE cm and post-training test: 2.4 ± 0.3 SE cm). In the post-training test, the stroke survivors did not change significantly either the systematic shift (*p* = 0.75) or the variability error (*p* = 0.54) with respect to the pre-training test (Fig. 6 panel a&b).

Specifically, during pre-training test all stroke survivors had a relevant retroversion of the pelvis and this did not change in the post-training test (Var_y_ -pre-training test: −0.9 ± 0.6 SE cm and post-training test: −0.5 ± 0.4 SE cm). Moreover, most stroke survivors (8 out of 10) had a slight shift toward impaired side during both the pre-training test and the post-training tests (Shift_x_: 0.3 ± 0.2 SE cm).

Considering this shift of the home position, we computed the direction and extent errors not only with respect to the real target positions as reported above, but also with respect to the target positions re-centered on actual initial position of the cursor in each trial. The absolute directional and the extent errors computed following this method confirmed the results described in the previous paragraph, since these indicators did not change significantly in the post-training test with respect to the pre-training test. Specifically, both measures did not change significantly either for the different target amplitudes (absolute extent error – pre-training test: L/2 = 4.0 ± 0.3 SE cm, L = 3.9 ± 0.3 SE cm, L + L/2 = 5.9 ± 0.6 SE cm and post-training test: L/2 = 3.9 ± 0.2 SE cm, L = 3.8 ± 0.3 SE cm, L + L/2 = 5.9 ± 0.4 SE cm; absolute directional error –pre-training test: L/2 = 22 ± 2.05 SE deg., L = 14.7 ± 0.9 SE deg., L + L/2 = 15 ± 0.9 SE deg. and post-training test: L/2 = 21 ± 1.4 SE deg., L = 14.3 ± 0.7 SE deg., L + L/2 = 15.2 ± 1.4 SE deg) or for the different target directions (absolute extent error - pre-training test: 4.4 ± 0.5 SE cm and post-training test: 4.3 ± 0.3 SE cm; absolute directional error - pre-training test: 16.2 ± 1.4 SE deg. and post-training test: 16.4 ± 0.9 SE deg).

### Individual performance

Most stroke survivors did not transfer the trained ability to the no visual feedback conditions. Only three out of ten had changes in the post-training performance with respect to their pre-training test. These subjects were different from the others also during training since they reduced not only the extent, but also the directional error. After training, one stroke survivor, P3, generalized the learned ability for both different target directions and displacement amplitudes. This subject had relevant changes post training for RE2 (different amplitudes: L/2: *p* = 0.03, L: *p* = 0.04, L + L/2: *p* = 0.001 and targets’ directions: *p* < 0.001), absolute extent error (different amplitudes: L/2: *p* < 0.001, L: *p* = 0.03, L + L/2: *p* = 0.01 and targets’ directions: *p* < 0.001) and absolute directional error (different amplitudes: L/2: *p* < 0.001, L: *p* < 0.001, L + L/2: *p* = 0.001 and targets’ directions: *p* < 0.001).

The subjects P2 and P4 had a significant decrease of the RE2 for the targets at the same distance from the home position, although in different directions (P2: *p* = 0.004 and P4: *p* = 0.02) with respect to the trained targets. Specifically, P2 reduced both the absolute extent error and the absolute directional error (*p* = 0.001 and *p* = 0.002, respectively) while P4 reduced only the extent error (*p* = 0.003). No relevant changes were found for the targets located at different displacement amplitudes.

## Discussion and conclusion

Subjects were seated on a force platform and were trained to reach targets positioned in three different directions at a fixed distance from a central target, by controlling a cursor with their CoP displacement. During training, subjects were provided with continuous visual feedback of the cursor.

Our goal was to study if chronic stroke survivors can lean the task and transfer the learned ability to the different conditions: (i) no visual feedback, (ii) other targets directions and (iii) different displacement amplitudes. Most chronic stroke survivors were able to perform the task, but they strongly relied on on-line visual feedback corrections and most of them did not transfer the learned ability to the no visual feedback condition.

We expected that stroke survivors were able to perform the required task and to improve their performance in presence of visual feedback. This supported by several studies on platform training for recovering balance while standing [16, 18, 22, 53, 54], sitting [20, 55] or during the transition between these two conditions [56]. In a broader view, this result is also in agreement with the finding that even if the control and execution of motor skills are impaired after stroke, the ability to learn those skills is not compromised [57].

However, stroke survivors strongly relied on visual feedback to complete the task during all training. They had relevant absolute directional error, but most of them did not cancel these errors in a predictive way as the training proceeded, they just corrected the second part of the cursor trajectory by using visual feedback. This finding is supported by the hypothesis that many subjects with hemiplegia have an excessive reliance on visual feedback [54] and this is observable since the acute stage [58, 59]. Thus, a training based only on continuous visual feedback may decrease in a significant manner the role of proprioceptive, tactile and vestibular feedback in the control of posture [60–62] even when these sensory modalities remain intact after the stroke. This negative effect may be even stronger in the case of proprioceptive impairment, precluding a progressive recovery of such important sensory channel. Thus, if subjects have postural control problems arising from these other feedback inputs, a training based on visual cues can lead to a reduced improvement of balance, with respect to a training based on visual cue deprivation [63].

This dependence on on-line feedback has also another important implication: it leads to modifying the behavior by relying more on on-line corrections than applying feedforward adjustments based on previous experience. Scheidt and Stoeckmann [64] observed a similar behavior for upper limb movements. They reported that stroke survivors assigned a significantly low weight to prior movement errors when planning subsequent reaching movements.

These findings suggest that stroke survivors have difficulties to form an internal model of the proposed task. The same conclusion was derived for the control of the contralesional arm during reaching movements: Takahashi and Reinkensmeyer [65] demonstrated that the hemiparesis stroke impairs the ability to implement internal models used for anticipatory control of arm movements.

In our experiment, when visual feedback was removed, stroke survivors had difficulty not only repeating the task, but also maintaining a correct sitting posture. Before training, stroke subjects had a marked retroversion of the pelvis and most of them tended to slightly shift their CoP toward the affected side. During training with VF they had a correct alignment of the spine, but after training they assumed again the same incorrect posture.

The fact that most stroke survivors when visual feedback was removed had difficulty maintaining a correct sitting posture or retaining information either of the direction or extent of their body shift, could predict also strong limitations for the translation to daily life activities of the postural control and balance skills learned with this VF training.

In this respect, some studies found improvement in falls prevention [56], in activities of daily living and gross motor functions [66]. However other works, investigating the effects of postural training in both standing [22, 31] and sitting [67] positions, highlighted a difficulty for stroke survivors to transfer to daily life activities the improvement obtained in solving the platform-based exercises. Also a Cochrane Review [53] concluded that providing feedback from a force platform do not improve balance and independence during functional activities.

However, stroke survivors were able to perform the required task and to improve their performance during training with visual feedback. These positive gains observed during training justify the integration of this technology-based protocol in a well-structured and personalized physiotherapy training. The combination of the two approaches may lead to functional recovery, as we observed for robotic and physical therapies in previous studies [68].

### Individual performance and limitations of the study

While the results presented and discussed were robust across our entire stroke subject population, there were important individual differences. One subject was not even able to improve task performance by using the visual feedback while another was able to transfer the learned ability to the task performed without visual feedback.

It is worth noticing that the three subjects, who to some extent transferred the learned ability to the no VF condition were the only ones among stroke survivors that also decreased the directional error with training.

There are several possible factors accounting for these differences, such as the location of the lesions [17, 69] and age. The subject who did not learn the task was the oldest one and had the lowest scores in the BBS and TIS clinical scales. However, further elaborations on this would not be warranted given the limited sample size of our subject population.

The small sample size of this study together with the absence of an age and a sex matched control group for the stroke survivors are the main limitations of this study. In a larger population, it would be possible and interesting to investigate the individual results taking into account the location of the lesion, the demographic data and the individual abilities of the subjects to proficiently use the somatosensory, vestibular, and visual inputs separately.

Further studies are necessary to verify if different protocols – for example based on intermittent or terminal feedback– could lead to more significant improvements for the chronic stroke population.

We cannot exclude that different results could be obtained with longer training over multiple days and with stroke survivors in the acute stage.

#### Clinical implication

This study suggests that a postural training based exclusively on continuous visual feedback could provide limited benefits for many stroke survivors, if administered alone i.e. not as a part of a well-structured and personalized physiotherapy training.

Since most subjects immediately after stroke have an impairment of proprioceptive sensory channels and thus are forced to exaggerate the importance of visual feedback [12] in the organization of purposeful actions, it would be important to design experimental protocols that are capable to provide effective on-line information of the impairment level of proprioceptive channels and, accordingly, can induce a gradual reduction of visual feedback in favor of the underused proprioceptive system.

Although it may appear that the main result of the study is to refute the ability of posture training programs focused on continuous visual feedback to provide robust clinical gains in postural recovery of stroke survivors, merely supporting the conclusion of the Cochrane review [53], we wish to emphasize that this is only part of the story. We wish instead to encourage therapists who are using this training protocol to continue using it, but with a clear understanding of its limits/drawbacks and in particular with a creative, patient-tailored combination of this technique with other (manual or technical) interventions that motivate the stroke survivors subjects to better attend the proprioceptive awareness of their body. In general, this attitude is consistent with the suggestion of a synergy between technology assisted therapy to physiotherapy in the treatment of stroke survivors [68].

## Abbreviations

ANOVA: Analysis of variance
BBS: Berg Balance Scale
cm: Centimeter
CoM: Center of Mass
CoP: Center of Pressure
deg.: degree
L: Length
ms: Millisecond
NJ: Normalized jerk index
NJ2: Normalized jerk index over the 2 s of movement
NSA: Nottingham Sensory Assessment Scale
RE2: Reaching error at 2 s
SE: Standard error
Shift: Systematic shift
TIS: Trunk Impairment Scale
Var: Variability error
VF: Visual feedback
